# Initial experience with surrounding en bloc transurethral resection of bladder tumor and simultaneous intravesical treating for non-muscle invasive bladder cancer

**DOI:** 10.1186/s12894-022-01140-2

**Published:** 2022-11-23

**Authors:** Lichen Teng, Changfu Li, Wentao Wang, Dechao Li

**Affiliations:** Department of Urology, Harbin Medical University Cancer Hospital, No.150 Haping Road, Harbin City, 150080 Heilongjiang Province China

**Keywords:** Surrounding en bloc resection, Transurethral resection of bladder tumor, Simultaneous intravesical chenmotherapy, Non-muscle invasive bladder cancer

## Abstract

**Purpose:**

The high recurrence rate after traditional transurethral resection of bladder tumor (TURBT) remains a challenge for management of non-muscle invasive bladder tumor (NMIBC). The aim of this study was to evaluate feasibility, efficacy and safety of surrounding en bloc resection using a general wire bipolar loop electrode and simultaneous intravesical chemotherapy.

**Methods:**

We retrospectively analyzed data of 111 consecutive patients with NMIBC treated from June 2018 to December 2021. These patients underwent conventional TURBT and immediate intravesical chemotherapy (n = 45) or surrounding en bloc TURBT and simultaneous intravesical chemotherapy in the Urology Department of Harbin Medical University Cancer Hospital, The former and latter were defined as the conventional TURBT group and the surrounding en bloc TURBT group, respectively. All patients were followed up from 6 to 40 months, with an average of 24 months. Demographic characteristics, location and number of tumors, perioperative and postoperative data, pathological results and recurrence were documented.

**Results:**

There were no significant differences in clinicopathological data between the conventional TURBT group (n = 45) and the surrounding en bloc TURBT group (n = 66). Operative time and complications associated with TURBT were comparable in the two groups. Recurrent tumors were found during follow-up in 2 (3.0%) of 66 patients in the surrounding en bloc group and 9 (20%) of 45 patients in the conventional group (*p* < 0.05). Lower urinary tract symptoms developed in 2 (3.0%) of 66 patients after surrounding en bloc TURBT and in 11(24.4%) of 45 patients after conventional TURBT (*p* < 0.05).

**Conclusion:**

Surrounding en bloc TURBT and simultaneous intravesical chemotherapy might significantly decrease the recurrence rate of NMIBC, and showed favorable safety and tolerability profiles. The general bipolar loop electrode was appropriate to complete the procedure.

**Supplementary Information:**

The online version contains supplementary material available at 10.1186/s12894-022-01140-2.

## Highlights

- Surrounding en bloc TURBT combining simultaneous intravesical chemotherapy might decrease recurrent rate in intermediate-term.
- Surrounding en bloc TURBT can be completed with general bipolar electrode loop.
- Simultaneous intravesical chemotherapy might increase efficacy and compromise uncomfortability of agents Surrounding en bloc TURBT can be completed with general bipolar electrode loop.
- The novel technique is feasible and safe for patients with NMIBC.

**Supplementary Information:**

The online version contains supplementary material available at 10.1186/s12894-022-01140-2.

## Introduction

Non-muscle invasive bladder cancer (NMIBC) accounts for 75% to 85% of all bladder malignancies [1]. At present, transurethral resection of bladder tumor [TURBT] still is the gold standard of care for patients with NMIBC [2]. Complete resection of all visible lesions is essential for both therapeutic and diagnostic targets. The conventional TURBT technique has been adopted worldwide, and many patients have been cured using this technique. To further decrease rates of recurrence and tumor progression, immediate postoperative instillation of chemotherapy following TURBT is also a standard recommendation [3, 4].

Conventional TURBT has some inherent technical limitations, such as piecemeal resection and frequent thermal injury to adjacent tissues. Moreover, it is very difficult to precisely evaluate pathological T stage on piecemeal tissues, because detrusor muscle in specimens is hard to identify. Cancer cells potentially seed into the bladder wall during the procedure, and may significantly increase the risk of recurrence [5]. To improve the oncological outcome of patients with NMIBC, several en bloc resection techniques have been developed [6–8]. However, some special instruments, like a special button electrode, waterjet, or laser are required [8, 9], and the general bipolar loop electrode has only been used in conventional TURBT, but not en bloc TURBT.

Generally, immediate instillation chemotherapy following TURBT should be completed within 24 h after surgery, which could reduce the risk of recurrence in patients with NMIBC [10]. To further increase the efficacy of instillation chemotherapy, we developed simultaneous intravesical instillation in which patients are administered immediate instillation chemotherapy of gemcitabine within 10 min after TURBT.

In the current study, we integrated en bloc resection of bladder cancer with the loop electrode and simultaneous instillation chemotherapy in patients with NMIBC. Here we report our initial experience, and we also compare oncological outcomes of the novel strategy with those of the conventional operation.

## Materials and methods

Data from 111 consecutive patients who were newly diagnosed with NMIBC by magnetic resonance imaging (MRI) scanning and diffusion-weighted imaging (DWI), and cystoscopic or cytological examinations and treated from June 2018 to December 2021 were analyzed. These patients underwent conventional TURBT and simultaneous intravesical chemotherapy (n = 45) or surrounding en bloc TURBT and simultaneous intravesical chemotherapy (n = 66) Inclusion criteria were primary NMIBC diagnosed with cystoscopic examination and MRI scanning and a tumor with a diameter of 0.5—4.0 cm. Postoperative pathological diagnosis was urothelial carcinoma. Exclusion criteria were recurrent NMIBC, muscle invasive bladder cancer, concomitant upper tract urothelial carcinoma, tumor at the bladder neck, and tumor with a diameter more than 4.0 cm. All patients underwent TURBT at the Department of Urology of Harbin Medical University Cancer Hospital. Surrounding en bloc and conventional TURBT were performed using F26.5 continuous flow resectoscopy and a general 12-degree bipolar electrode loop (ESG-400, Olympus, Japan). The cutting and coagulation mode were set at 200 and 120 W, respectively. The study protocol was approved by the Ethical Committee of Harbin Medical University Cancer Hospital. All patients signed informed consent before the procedure. Clinical data from these patients were collected and analyzed retrospectively.

### Surgical procedure

**Fig. 1:**
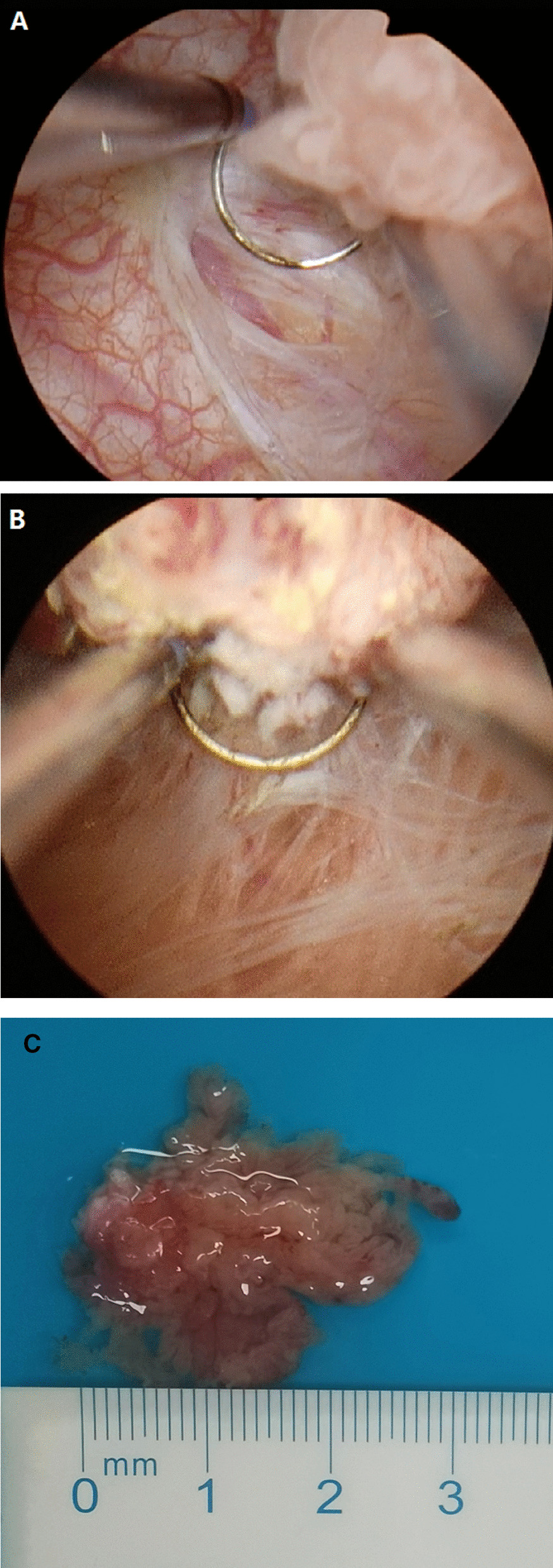
The steps of surrounding en bloc TURBT using bipolar electrode loop. **A** Initially, mucosa surrounding the tumor at 1.0—1.5 cm away from tumor were cut into muscle layer, and the muscle layer was clearly seen. **B** The intact tumor with base was lifted up on the bladder wall along the muscle layer. **C** The tumors were removed in en bloc fashion from the bladder cavity

Patients were placed in the dorsal lithotomy position after induction of general anesthesia. To avoid missing the concomitant tumor, and to confirm the position of tumors, complete cystourethroscopy with F-26 resectoscope (Olympus) was completed again before resection. During the procedure irrigation was with 0.9% saline. Initially, normal mucosa at 1.0—1.5 cm away from the tumor was excised using the surrounding tumor technique. The muscle layer was reached, and muscle fibers were clearly seen. Next, blunt push combined with electric resection was moved forward to the base of the tumor along the muscle layer (Fig. 1A and B). Based on the location of the tumor, the base of tumor was lifted or pushed using the bipolar loop electrode under clear vision, and the intact tumor was removed from bladder walls (Fig. 1C). The direction of the loop electrode was frequently regulated according to the tumor shape. For multiple tumors, the bladder wall was divided using the electrode loop into some round areas each of which contained some lesions. A similar technique was used to perform surrounding en bloc TURBT. To prevent potential seeding, the tumor was not touched by the loop electrode during operation. The specimen was immediately removed through a resectoscope sheath once resection was completed (Additional file 1).

### Postoperative simultaneous intravesical chemotherapy

All patients in the surrounding en bloc TURBT group and the conventional TURBT group received simultaneous intravesical chemotherapy. Gemcitabine 1.0 g was dissolved in 40 mL 0.9% saline. An F-18 Foley catheter was placed into the bladder once the resectoscope sheath was removed, then gemcitabine solution was immediately instilled into the bladder cavity within 10 min after TURBT in the operating room At same time, the Foley catheter was clipped for 1 h. Next, patients were administered intravesical chemotherapy with gemcitabine weekly for 8 weeks and monthly thereafter, for 10 months. Patients were followed up every 3 months using MRI scanning, cystoscopic and cytological examinations.. We performed biopsy during cystoscopic examination if evidence was found of suspected recurrence.

### Statistical analysis

Non-parametric Mann–Whitney and chi-square tests were used to analyze continuous and categorical data, respectively. A P-value less than 0.05 was considered a statistically significant difference. All analyses were performed with SPSS software package (SPSS 19.0, Chicago, IL, USA).

## Results

According to the inclusion criteria,66 patients underwent the surrounding en bloc TURBT and 45 patients underwent conventional TURBT,. There were no significant differences in demographic characteristics, age, tumor diameter, number of tumors, T stage and grade between the two groups (*p* > 0.05) (Table 1). A single lesion was found in 36 (54.5%) of 66 patients in the surrounding en bloc TURBT group and 27 (60.0%) of 45 patients in the conventional TURBT group. Multiple lesions were found in 30 (45.5%) of 66 patients and 18 (40.0%) of 45 patients, respectively. The tumor diameter ranged from 0.5 to 4.4 cm (2.25 ± 0.96 cm) in the surrounding en bloc gr in the surrounding en bloc group and 0.8 to 4.5 cm (1.91 ± 0.95 cm) in the conventional group, and there was no statistically significant difference in tumor diameter between the two groups.

**Table 1:** Baseline clinical characteristics of patients

Variables	Surrounding en bloc TURBT (n = 66)	Conventional TURBT (n = 45)	*P* value
*Gender*			
Male	46 (69.7%)	38 (84.4%)	0.078
Female	20 (30.3)	7 (15.6%)	
Age (Year)	61.00 ± 12.40	61.93 ± 10.94	0.222
Diameter of tumor (cm)	2.25 ± 0.96	1.91 ± 0.95	0.067
< 3 cm	51 (77.2%)	36 (80.0%)	
> = 3 cm	15 (22.8%)	9 (20.0%)	
Number of tumor			0.672
Single tumor	36 (54.5%)	27 (60.0%)	
Multiple tumor	30 (45.5%)	18 (40.0%)	
T stage			0.570
Ta	39 (59.1%)	29 (64.4%)	
T1	27 (40.9%)	16 (35.6%)	
Concomitant CIS	0	0	
Presence of muscularis propria	63 (95.5%)	36(80.0%)	0.010
Grade			0.925
Low grade	45 (68.2%)	34 (75.6%)	
High grade	21 (31.8%)	11 (24.4%)	
Risk groups			0.351
Low	22 (33.3%)	16 (35.6%)	
intermediate	26 (39.4%)	18 (40.0%)	
High	18 (27.3%)	11 (24.4%)	

The results of perioperative data and postoperative follow-up data in both patient group are summarized in the Table 2. Follow-up time in the surrounding en bloc and the conventional TURBT ranged from 6 to 48 months, and there was no significant difference in follow-up time between the two groups. During follow-up, only two patients in the surrounding en bloc group, compared with nine patients in the conventional group presented recurrent tumor. In the surrounding en bloc group, multiple lesions on anterior wall were found in one patient 4 months postoperatively and a single recurrent tumor located on the bladder neck was found in one patient 14 months postoperatively. Recurrence-free survival (RFS) in surrounding en bloc TURBT group was 97.0%, while RFS in the conventional TURBT group was 80.0% (*p* = 0.003).

**Table 2:** Perioperative complications and oncological outcome

Variables	Surrounding en bloc TURBT (n = 66)	Conventional TURBT (n = 45)	*P* value
Operation time (min)	30.00 ± 7.07	38.53 ± 18.27	0.374
Bladder perforation	0 (0%)	0 (0%)	-
Obturator reflex	6 (21%)	12 (26%)	0.422
Postoperative length of stay (day)	3.4 ± 1.9	4.6 ± 2.0	0.014
Postoperative catheterization (day)	5.5 ± 1.9	6.6 ± 2.0	0.015
Follow-up time (month)	22.8 ± 9.0	25.7 ± 6.9	0.065
Cumulative recurrence	2	9	0.003

None of the patients in either group needed readmission for perioperative complications, such as post-operative bleeding and vesicle perforation. All patients who received simultaneous intravesical instillation showed favorable tolerability. Lower urinary tract symptoms, such as frequent, urgent micturition and dysuria, occurred in 2 (3.0%) of 66 patients in the en bloc group and 11 (24.4%) of 45 patients in the conventional group after simultaneous intravesical chemotherapy, respectively (*p* = 0.032). These symptoms were reported by patients postoperatively within 1 to 3 days.

## Discussion

Our preliminary experience demonstrated that the novel technique of surrounding en bloc resection with the general bipolar electrode loop combined with simultaneous intravesical instillation of chemotherapy was feasible and safe. Moreover, fewer cases of recurrence were found in patients who underwent the novel surrounding en bloc procedure than in those who underwent the conventional procedure. No perforation or delayed bleeding was observed in patients in the two groups. Initial electrocoagulation or resection of surrounding tumor, not touching the tumor with the loop electrode and en bloc resection along deep muscle layer may have contributed to low recurrent rates and tumor progression, which would maximally decrease intravesical spread of tumor cells. Many related factors contributed to the outcome, such as more exposure of a definite deep muscle layer. Compared with traditional piecemeal TURBT, mechanical push combined with thermal energy was used to substantially enucleate the tumor from the deep muscle layer. Moreover, the technique also protected the deep muscle layer from thermal injury, contributed to precise pathological evaluation. Furthermore, simultaneous instillation of chemotherapeutic agents also induced the maximal cytotoxic effect on remaining tumor cells prior to potential direct implantation [11–13]. This also can prevent patient discomfort from chemotherapeutic agents because intravesical instillation was during the postoperative anesthetic period.

Traditional TURBT remains the mainstream surgical procedure for NMIBC, but, due to absence of muscularis propria in most specimens and an indefinite scope of resection, the recurrence rate remains high (from 50 to 70%) [14]. The possible reason may be that multiple pieces of specimens are prone to spread active tumor cells. According to the recommendations of the EAU guidelines, a second TURBT should be performed within 2 to 6 weeks when the initial TURBT is incomplete, there was no muscle in specimen in the initial TURBT, or where a stage T1 tumor is detected. Compared with traditional TURBT, surrounding en bloc resection decreases possibility of incomplete TURBT and the lack of muscle, and even for most patients with stage T1 tumor, a second TURBT has not been required. The novel technique may provide sufficient size and depth of resection and accurate pathological diagnosis.

Although an en bloc resection technique for NMIBC has been reported, in our study a general bipolar electrode loop and resectoscope without other special equipment were used to perform surrounding en bloc resection of bladder tumor. This differs from previous studies with regard to the instruments used, such as J shape electrode or button electrode [6, 15, 16]. Additionally, laser also was used in en bloc resection of bladder tumor, but a new energy platform should be required. Initially, we coagulated mucosa and vessels surrounding the tumor, vertically incised mucosa to the deep muscle layer, and then used the fine loop to push or resect the layer. Another retrograde en bloc technique was reported [17], and it is feasible for solitary and small lesions However, a large tumor and multiple tumors the technique may be not suitable, because the outline of tumors may interfere with the vision of the urologist during resection.

Immediate intravesical chemotherapy following TURBT is considered an effective approach to reduce risk of recurrence [18, 19], however, the optional timing of intravesical therapy remains unclear [20]. Because previous reports have shown that tumor cells are firmly implanted within the first few hours after TURBT, intravesical chemotherapy should be administered as early as possible [21, 22]. This is why we used simultaneous instillation of intravesical chemotherapy. Our study provided evidence of the benefit of simultaneous instillation of chemotherapeutic agents in NMIBC. Favorable outcomes inspired us to widely use this therapeutic approach after surrounding en bloc TURBT. The results suggest that sporadic active tumor cells may be killed by early administration of cytotoxic chemical agents. Patients have not experienced any uncomfortable response during instillation because they are in the postoperative anesthetic period, and the results of our study showed that simultaneous intravesical chemotherapy was well tolerated. According to a previous report and the EAU guidelines on NMIBC, immediate intravesical instillation should be performed within 24 h after TURBT [23, 24]. In our study, we observed that most patients do not tolerate instillation of pirarubicin or gemcitabine more than 30 min postoperatively, which we suppose may have compromised the efficacy of these agents.. To overcome these disadvantages, we developed the simultaneous intravesical therapy technique.

Our study has some limitations. Because it was a retrospective analysis, we performed the novel surrounding en bloc technique and conventional TURBT at different periods of time. Tumor on the bladder neck is not applicable for surrounding en bloc TURBT. There was a lack of the postoperative cytological evidence in the study. The small size of the patient groups and the short follow-up period make it difficult to draw a conclusion with regard to survival. A prospective, randomized, controlled trial is needed in the future.

## Conclusions

The results of the present study suggest that surrounding en bloc TURBT and simultaneous intravesical therapy with a general bipolar electrode loop can be widely adopted by urologists. It will benefit most patients with NMIBC in terms of reducing recurrence, even for T1 bladder cancer. The novel strategy was feasible, effective and safe. We suggest that surrounding en bloc TURBT with simultaneous chemotherapy will be the new standard for treating NMIBC in the future.

## Supplementary Information

**Additional file 1. Video S1**. Surrounding tumor en bloc TURBT was performed for 2.0 cm diameter tumor that located on the left lateral wall of bladder triangle in a patient. We cut surrounding mucosa at 1.0 cm away from tumor using general bipolar electrode loop. Resection deepened to muscle layer, and the tumor with base muscle was resected in en bloc way along the normal layer. The tumor was minimally touched during resection. Finally, the tumor was removed in one piece through outer sheath of resectoscope.

## Data Availability

The datasets used or analyzed during the current study are available from the corresponding author on reasonable request.
